# The Role of Testosterone and Gibberellic Acid in the Melanization of *Cryptococcus neoformans*

**DOI:** 10.3389/fmicb.2020.01921

**Published:** 2020-08-13

**Authors:** Jamila S. Tucker, Tiffany E. Guess, Erin E. McClelland

**Affiliations:** ^1^University of Kentucky, Lexington, KY, United States; ^2^Department of Biology, Middle Tennessee State University, Murfreesboro, TN, United States; ^3^Vanderbilt University Medical Center, Nashville, TN, United States

**Keywords:** *Cryptococcus neoformans*, melanization, testosterone, gibberellic acid, pantothenic acid

## Abstract

*Cryptococcus neoformans*, a spore-producing pathogenic yeast, affects immunocompromised individuals causing meningoencephalitis. Once *C. neoformans* is introduced via the respiratory tract, it is engulfed by macrophages and other phagocytes. One of *C. neoformans’s* primary virulence factors is the pigment melanin, which is formed in the cell wall and protects the yeast against UV radiation and oxidizing agents produced by macrophages during phagocytosis. To better understand the observed sex bias (3:1; male:female) in *C. neoformans* infections, the phenotype of various virulence factors was determined in the presence of exogenous sex hormones. *C. neoformans* melanized faster in the presence of testosterone than it did in the presence of estradiol. Using a combination of RNA sequencing analysis and ELISA results, we identified a growth hormone, gibberellic acid (GA), produced in *C. neoformans* that was highly upregulated in the presence of testosterone. A variety of knockout strains of genes involved in the GA biosynthesis pathway showed significantly reduced melanization in the presence of testosterone. Additionally, inhibitors of GA also reduced melanization in the presence of testosterone. Thus, these data suggest that the gibberellic biosynthesis pathway is involved in melanization in *C. neoformans*, and the melanization difference observed in the presence of testosterone may be due to increased production of GA, which may partly explain the sex bias observed in *C. neoformans* infections.

## Introduction

Decades of epidemiological data indicate an approximately 3 male:1 female sex bias in *C. neoformans* infections (Campbell, 1966; Edwards et al., 1970); however, the reasons for these differences remain controversial (Emmons, 1955). Data from several studies indicate that disparities in infection rates have a biological, and not solely environmental, basis (Chen et al., 2000; Du et al., 2015). For example, we previously showed the steroid hormone, testosterone, increases extracellular release of the polysaccharide capsule (McClelland et al., 2013), a key virulence factor and immunomodulator of *C. neoformans*, which suggests that a testosterone-rich environment could be contributing to an increase in virulence in cryptococcosis. In patients with HIV and cryptococcal meningitis, males have higher levels of CD4^+^ T cells, but mortality rates are higher compared to females (McClelland et al., 2013). Additionally, males are more susceptible to primary cutaneous cryptococcosis than females (Du et al., 2015); while a study completed in Australia indicated that immunocompetent males also have an increased risk of *C. neoformans* infections, due to unknown reasons (Chen et al., 2000). This could be due to a T cell deficit in immunocompetent males, as healthy males have lower *ex vivo* T cell percentages during a *C. neoformans* infection than females (Guess et al., 2019). We hypothesize that this may partly explain the sex susceptibility observed during infections (Guess et al., 2019). To determine if steroid hormones were also contributing to this sex difference, we measured virulence factor expression in the presence of these hormones and assayed the phenotype of various virulence factors.

Melanin is produced via two pathways in fungi: the 1,8-dihydroxynaphthalene (DHN) pathway and the L-3,4-dihydroxyphenylalanine (L-DOPA) pathway (Eisenman and Casadevall, 2012). *C. neoformans* develops melanin via the L-DOPA pathway, using L-DOPA and/or a wide variety of phenolic precursors as exogenous substrates, which is catalyzed by laccase to form melanin (Eisenman et al., 2011). As in other pathogenic fungi, melanin production aids *C. neoformans* survival in the environment against harsh environmental conditions and UV radiation (McFadden and Casadevall, 2001). However, inside the host, melanization benefits *C. neoformans* in multiple ways. Melanin production has been associated with the reduction of tumor necrosis factor-α and lymphoproliferation, which allows *C. neoformans* to avoid phagocytosis (Casadevall et al., 2000). If phagocytosed, melanin can also protect the yeast from oxidizing agents produced by macrophages, which allows *C. neoformans* to avoid oxidative damage and replicate inside the macrophage (Wang et al., 1995). Melanization is cell-density dependent and controlled by quorum sensing, a mechanism by which microbes release chemical signals called quorum sensing molecules (QSMs) that increase in concentration as a function of cell density (Bateman et al., 1990). Once QSMs are at a specific concentration, changes in gene transcription induce a certain phenotype (Miller and Bassler, 2001). The discovery of this mechanism in *C. neoformans* is fairly recent. A study conducted by Albuquerque et al. (2013), identified the QSM pantothenic acid (PA) in *C. neoformans* using gas chromatography, and it is hypothesized that PA, a precursor to coenzyme A (White et al., 2001), acts as a QSM and contributes to melanization in *C. neoformans* (Albuquerque et al., 2013).

We identified a difference in melanin production in the presence of testosterone versus estrogen. A combination of RNA sequencing analysis and enzyme-linked immunosorbent assay (ELISA) results suggested that this melanization difference in the presence of testosterone could be due to the production of gibberellic acid (GA). GA is a growth hormone most commonly found in plants and is required for the growth rate of seedlings (Hedden et al., 2001). This acid, first identified in the pathogenic fungi *Gibberella fujikuroi*, functions in cell growth (McInnes et al., 1977) and has since been found in a number of other fungi (Salazar-cerezo et al., 2018). To date, the production of GA has not been identified in *C. neoformans*. We hypothesize that this molecule is upregulated in the presence of testosterone, increasing the rate of melanization of *C. neoformans*, which could be contributing to the increased disease observed in men.

## Materials and Methods

### *C. neoformans* Strains

All experiments were conducted using the wildtype *C. neoformans* strain, H99S (serotype A) (Perfect et al., 1980; Janbon et al., 2014), unless otherwise stated. H99S was grown from frozen stock in yeast peptone dextrose (YPD) broth (Fisher Scientific; Hampton, NH, United States) for 24–36 h (log-phase) in an orbital incubator at 37°C for all treatments. After incubation, the cells were washed in phosphate-buffered saline (PBS) three times and resuspended in PBS. Cells were counted and diluted to a final concentration of 1 × 10^6^ cells/mL. Clinical isolates obtained from the cerebrospinal fluid of HIV^+^ patients with cryptococcal meningitis in Botswana (Bisson et al., 2008) were used in initial experiments to determine if melanization varied from the wildtype. *C. neoformans* knockout (KO) strains of genes identified from the RNA sequencing data were a kind gift from Dr. Hiten Madhani (University of California, San Francisco) and did not show a melanization phenotype (Liu et al., 2008).

### Growing *C. neoformans* on L-DOPA Agar Plates and Liquid Medium

**FIGURE 1:**
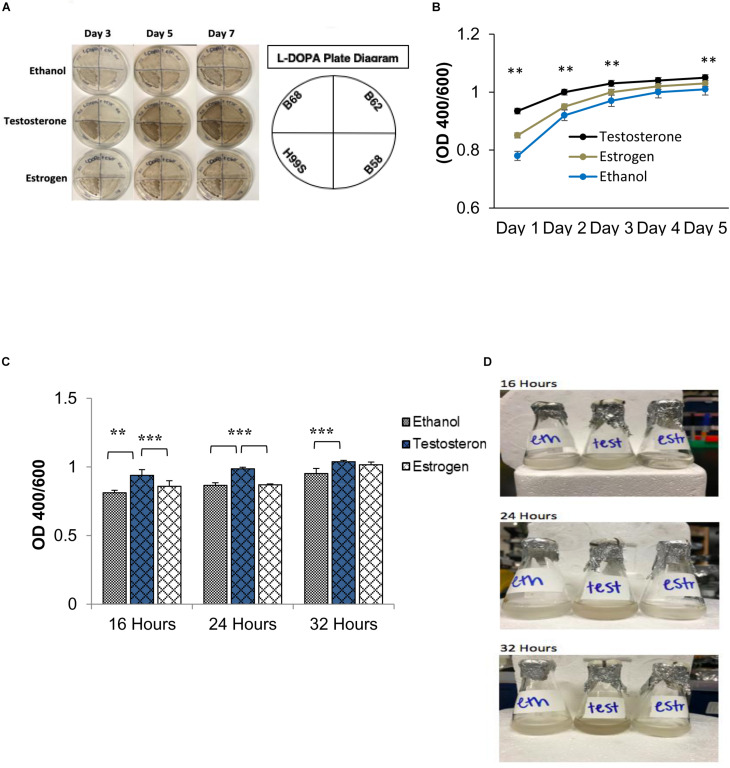
Melanization of *C. neoformans* on L-3,4-dihydroxyphenylalanine (L-DOPA) agar plates and liquid medium in the presence of steroid hormones (estradiol, 400 pg/mL; testosterone, 10 ng/mL). **(A)** Melanization of *C. neoformans* clinical isolates grown for 7 days on L-DOPA agar plates supplemented with steroid hormones. Images were taken on days three, five, and seven. **(B)** Melanization of H99S grown for 5 days in L-DOPA medium supplemented with steroid hormones and measured at an optical density of 400 and 600 nm. Data is representative of three independent experiments. Statistical significance is represented as follows: ^∗∗^*p* < 0.001 comparing testosterone to ethanol and estradiol, using linear regression analysis. **(C)** Melanization of *C. neoformans* in conditioned media supplemented with steroid hormones. Absorbances were measured at an optical density of 400 and 600 nm. Error bars represent the standard deviation. Data is representative of three independent experiments. Statistical significance is represented as follows: ^∗∗^*p* < 0.002, ^∗∗∗^*p* < 0.0002 using MANOVA with simple contrasts, comparing testosterone to estrogen or ethanol. **(D)** Pictures of H99S melanized cells from **(C)** at 16, 24, and 32 h. Test, Testosterone; Estr, Estrogen (Estradiol); Eth, Ethanol.

H99S and clinical isolates were grown and prepared as stated above. Using a modified melanization protocol, exogenous hormones were added at physiological concentrations (17β-estradiol: 400 pg/mL (Calbiochem, San Diego, CA, United States; testosterone: 10 ng/mL, Sigma Aldrich, St. Louis, MO, United States) to the 1 mM L-DOPA (0.1982 g, Sigma Aldrich, St. Louis, MO, United States) liquid minimal media and agar plates (Eisenman and Casadevall, 2012). Ethanol was used as the diluent for both steroid hormones and was therefore used as a vehicle control. Three clinical strains and H99S (1 × 10^6^ cells per strain) were streaked onto each plate (one per quadrant, see Figure 1A). The plates and flasks were wrapped in foil for protection from light and stored in a 37°C incubator for 5 days (liquid media) or 7 days (plates). One milliliter was removed from the flasks every 24 h for an absorbance measurement. An optical density of 400 and 600 nm (to correct for cell density) was determined as by Baker et al. (2007). On days three, five, and seven, images were taken of the L-DOPA plates to document the amount of melanization.

### Conditioned Media Using Exogenous Hormones

Conditioned media was made using the L-DOPA liquid media experiment described above. *C. neoformans* was grown in liquid L-DOPA media supplemented with steroid hormones for 7 days to allow melanization. On day seven, melanized cultures were centrifuged at 750 × *g* for 5 min to pellet cells and supernatants were collected. Supernatants were plated to determine that all cells from the culture were removed. A fresh *C. neoformans* culture was started on day six and prepared as stated above. In three flasks treated with physiological concentrations of testosterone, estrogen, or a 1:100 dilution of ethanol, respectively, new L-DOPA media and the collected supernatants of the melanized cultures were added in a 1:1 fashion with fresh *C. neoformans* cells. The flasks were wrapped in foil for protection from light and incubated at 37°C in an orbital shaker at 150 RPM. Absorbance measurements at an optical density of 400 and 600 nm were taken at three timepoints, 16, 24, and 32 h, and the pH was tested for each hormone treatment using pH tests strips.

### Testosterone and Estrogen Dose-Response

H99S was grown to log-phase, as stated above. Five concentrations of exogenous testosterone (0, 0.5, 2, 5, and 10 ng/mL) and estradiol (0, 100, 200, 400, and 10 ng/mL) were added to the cells and conditioned L-DOPA media, respectively. The flasks were protected from light and incubated for 32 h in a 37°C orbital shaker at 150 RPM. An absorbance measurement at an optical density of 400 and 600 nm was taken at three timepoints, 16, 24, and 32 h.

### RNA Isolation

H99S was grown to log-phase, as stated above. Three flasks were treated with testosterone, estrogen, or ethanol, respectively. Cells, hormones, and L-DOPA liquid media were all added to the flasks at a final volume of 10 mL. The flasks were protected from light and placed in a 37°C orbital shaker for 3 days. After incubation, cells were centrifuged at 750 × *g* for 5 min, counted, and diluted to a final concentration of 2 × 10^8^ cells for RNA isolation using an RNeasy Midi Kit (Qiagen, Hilden, Germany). Genomic DNA contamination was removed from the total RNA using a Message Clean Kit (GeneHunter, Nashville, TN, United States), and the RNA concentration was measured using a NanoDrop 2000 Spectrophotometer (Thermo-Scientific, Waltham, MA, United States).

### Quantitative Reverse Transcription Polymerase Chain Reaction (qRT-PCR)

qRT-PCR was performed using RNA isolated from *C. neoformans* at day three of melanization. cDNA was prepared using the QuantiNova^TM^ Reverse Transcription kit (Qiagen, Hilden, Germany) using 1 μg of isolated RNA. cDNA, primers, and SYBR green were added to a 96-well PCR plate that amplified the products using a CFX96 Real-time PCR Detection System (BioRad Laboratories, Hercules, CA, United States). Each cDNA treatment was performed in quadruplicate and normalized using the geometric mean of the housekeeping genes *UBC6* and *TFC1*. Fold changes were determined using the 2^–ΔΔC′T^ method, as described by Livak and Schmittgen (2001).

### cDNA Preparation for RNA Sequencing

cDNA libraries for RNA sequencing (RNAseq) were prepared from 1 μg of isolated RNA using the NEBNext Ultra^TM^ Directional RNA Library Prep Kit for Illumina (New England BioLabs, Ipswich, MA, United States), the NEBNext Multiplex Oligos for Illumina Index Primers and the NEBNext Poly(A) Magnetic Isolation Module (New England BioLabs, Ipswich, MA, United States). Sequencing (75 bp, paired end with 22–57 million reads) was completed by HudsonAlpha Genomic Sequencing Lab (Huntsville, AL, United States) using the HiSeq 2500 system.

### RNAseq Analysis

RNAseq analysis was completed using two analytical platforms, CyVerse and Galaxy. Quality check was completed on each paired-end read using FASTQC (version 0.11.5 multi-file). Due to issues with STAR (version 2.5.3a-index-align), the paired-end reads were aligned using HISAT2 (version 2.1-index-align) to a *C. neoformans* (H99S) reference genome found using ENSEMBL.

Bam files produced from the HISAT2 (version 2.1-index-align) alignment were utilized in two ways. In Galaxy, the bam files were used to quantify the number of reads using a genome annotation file of *C. neoformans* found in ENSEMBL. MultiJoin (also in Galaxy) was used to join the output files of featureCounts into a single tabular file. This file was utilized in R studio using the edgeR differential expression package. To determine the similarities among replicates in the treatment groups (testosterone, estrogen, and ethanol) and to ensure any outliers would be removed, multidimensional scale plots (MDS plot) and a cluster dendrogram were utilized.

Once cluster analysis was complete, the reads were aligned into any potential transcripts using StringTie and the genome annotation (version 1.3.3) (CyVerse). Transcripts for all replicates in the treatment groups were then merged into a single gtf file using StringTie-Merge (version 1.3.3). Lastly, CuffDiff2 (version 16-way max) was utilized to compare the genome alignments (bam files) and the transcript alignments (gtf files) for gene expression changes that were significantly different. This output file was used to determine any genes that were upregulated when *C. neoformans* was in the presence of testosterone compared to the other treatment groups.

### ELISA to Detect Pantothenic and Gibberellic Acid

GA (Sigma Aldrich, St. Louis, MO, United States) and PA (MP Biomedicals, Santa Ana, CA, United States) concentrations in *C. neoformans* supernatants during melanization were determined using ELISAs. A standard curve for both PA (9.76–1250 ng/mL) and GA (62.5–500 ng/mL) were utilized (Gonthier et al., 1998; Gokani and Thaker, 2002). A primary rabbit polyclonal antibody (Lifespan Biosciences, Seattle, WA, United States) for both GA (1/400 dilution) and PA (1/600 dilution) was added to the samples, and they were incubated in a 37°C water bath for 1 h. After incubation the samples were transferred to a 96 well flat-bottom microplate (Greiner Bio-One, Kremsmunster, Austria) in duplicate and incubated again at 37°C for 1 h. Using a microplate washer (BioTek, Winoski, VT, United States), the wells were washed with PBS + 0.5% Tween 20 wash buffer. A secondary donkey anti-rabbit horseradish peroxidase polyclonal antibody (1/4000 dilution, Southern Biotech, Birmingham, AL, United States) was added to each well and the plate was incubated for another hour at 37°C. After incubation, the plate was washed using PBS + 0.5% Tween 20. The 3,3′,5,5′-tetramethylbenzidine substrate (TMB, Thermo Fisher Scientific, Waltham, MA, United States) was added to each well, and the plate was incubated for 30 min at room temperature. After incubation, 2 N sulfuric acid stop solution was added to each well. The absorbance of each sample was measured using the CLARIOstar Plus Microplate Reader (BMG LABTECH, Offenburg, Germany) at 450 nm.

### Phenotypic Testing of KO Strains in Conditioned Media

KO strains (a kind gift of Dr. Hiten Madhani, UCSF) of *C. neoformans* genes identified in the RNA sequencing and H99S were prepared as stated above. Following similar methods of the initial L-DOPA liquid media experiments (above), exogenous testosterone was added to all flasks containing strains and the flasks were incubated for 5 days. One milliliter was removed from the flasks every 24 h for an absorbance measurement at an optical density of 400 and 600 nm.

### Growth Curve of H99S in Steroid-Hormone Supplemented L-DOPA Medium

H99S cells were prepared as stated above, with the exception of a starting cell concentration of 1 × 10^5^ cells/mL. Following similar methods of the initial L-DOPA liquid media experiments, exogenous steroid hormones were added to all flasks containing H99S and incubated in a 37°C orbital shaker at 150 RPM for 9 h. After the 9 h incubation, one milliliter was removed from the flasks every 6 h for an absorbance measurement at an optical density of 600 nm. Measurements continued until all cultures reached an absorbance of 1.00 or stopped growing.

### Conditioned Media Using Exogenous QSMs

To test the effects of PA and GA on melanization, the conditioned media experiment (above) was modified. Using snap-cap tubes, a variety of additives/inhibitors were added to tubes containing steroid hormone supplemented conditioned L-DOPA media and *C. neoformans* cells. The additives/inhibitors included: 3 μg/mL GA (Sigma Aldrich, St. Louis, MO, United States), 2 × 10^–6^ M chlormequat chloride (GA inhibitor, Sigma Aldrich, St. Louis, MO, United States), 10 μM PA (MP Biomedicals, Santa Ana, CA, United States), and 1 mg/mL DL-Serine (PA inhibitor, Alfa Aesar, Harverhill, MA, United States). The tubes were wrapped in foil for protection from light and incubated at 37°C in an orbital shaker at 150 RPM. An absorbance measurement at an optical density of 400 and 600 nm were taken at three timepoints, 16, 24, and 32 h, and the pH was tested for each hormone treatment using pH tests strips.

### Fungal Burden and Survival

To determine fungal burden, adult male and female BALB/c mice (6 of each per sex, 6–8 weeks old obtained from Jackson Laboratories) were infected via intratracheal injection with 1 × 10^4^ CFU of H99S. Mice were injected intraperitoneally with a 2.5:1 mix of ketamine:xylazine to anesthetize them (5–10 mg/kg) prior to surgery. For the procedure, mice were placed on their back, their neck area was cleaned with alcohol and a small incision was made over the thyroid. The skin was gently pulled aside, and 50 μL of H99S was injected directly into the trachea using a bent tuberculin needle. The incision was closed using VetBond and the mice were kept warm and observed closely until they regained consciousness. Mice were euthanized using carbon dioxide overdose at day 7 post-infection and the spleen, lungs and brain were removed and homogenized. Homogenates were diluted and plated on YPD plates for 2 days at 37°C and colonies were counted to determine fungal burden.

To determine survival, adult male and female BALB/c mice (10 of each per sex, 6–8 weeks old obtained from Jackson Laboratories) were infected via intratracheal injection with 1 × 10^4^ CFU of H99S, as above, and observed over the course of infection. Any mouse in a moribund state and/or distress was euthanized using carbon dioxide overdose to avoid unnecessary suffering. Mice were monitored daily for mortality and morbidity and deaths or dates euthanized were recorded.

### Conditions That Mimic the Host Lung

H99S cells (1 × 10^5^ cells/mL) were added to Dulbecco’s modified Eagle media containing testosterone, estradiol, or ethanol and incubated at 37°C + 5% CO_2_ for 18 h, as described in Charlier et al. (2005). After incubation, supernatants were collected, centrifuged to remove *C. neoformans* cells, and stored at −20°C until used to assess PA and GA concentrations by ELISA (see section “ELISA to Detect Pantothenic and Gibberellic Acid”).

### Statistical Analysis

Melanization studies in liquid L-DOPA media over 5 days and dose-response curves were analyzed using linear regression analysis. Conditioned media experiments and serial dilutions were analyzed using multivariate analysis of variance (MANOVA) with simple contrasts. ELISA data were analyzed using Tukey Kramer’s honest significant difference for multiple comparisons and MANOVA with simple contrasts. qRT-PCR data were analyzed using the 2^–ΔΔC′T^ method (Rao et al., 2013) for relative gene expression analysis. RNA sequencing data were analyzed using edgeR differential expression analysis using R studio (Boston, MA, United States). Fungal burden data and survival were analyzed using Wilcoxon Ranks Sums test. Statistical analysis was conducted using JMP, version 14 (SAS Institute, Cary, NC, United States). *p* < 0.05 was considered significant.

## Results

### Variation in the Melanization of *C. neoformans* on Steroid Hormone-Supplemented L-DOPA Agar Plates, Liquid Medium, and Conditioned Medium

Initial data suggested that the melanization rate of *C. neoformans* could be influenced by steroid hormones. As shown in Figure 1A, a number of *C. neoformans* clinical isolates were grown on L-DOPA agar plates supplemented with steroid hormones for 7 days to test this hypothesis. Visually, there was a clear difference in melanization when *C. neoformans* was in the presence of testosterone. To determine if testosterone was perhaps a substrate for melanization, cells were grown on plates containing testosterone or estrogen without L-DOPA; the cells did not melanize in the presence of either hormone (data not shown). Thus, to derive quantitative data of this difference, the wild-type H99S *C. neoformans* strain was grown in liquid L-DOPA medium supplemented with steroid hormones or ethanol as a vehicle control for 5 days. Experiments to test for an effect of ethanol did not show any difference in fungal growth or melanization (data not shown). Each day the absorbance at an optical density of 400 and 600 nm (to correct for cell density) was measured. An increase in melanization when *C. neoformans* was in the presence of testosterone (*p* < 0.001) became apparent from day one and this trend continued throughout day five, except for day four, where there was no significant difference between any of the treatments (Figure 1B).

The regulation of gene expression in response to population density is a phenomenon known as quorum sensing (Nealson and Hastings, 1979). Because we hypothesized that a QSM might be responsible for the observed difference in melanization, to determine if *C. neoformans’* known QSM, PA, affected melanization when steroid hormones were present, a conditioned media experiment was conducted. Conditioned media is the supernatant of cells grown in media for 7 days; thus any secreted QSM that increased growth rate would be found in this supernatant. If *C. neoformans* was producing a QSM that affected melanization, an increase in melanization when conditioned media was used would be expected. To test this hypothesis, the wild type H99S strain was grown in conditioned media for 32 h. The absorbance at an optical density of 400 and 600 nm was measured at three time points, 16, 24, and 32 h. The pH of each treatment was also tested using pH test strips and showed no difference in pH (data not shown). In the previous 5-day experiments, the variation in melanization occurred at day three; however, when conditioned media was used, this variation was seen at 16 h (Figures 1C,D). There was also a significant increase in melanization (*p* < 0.002) when *C. neoformans* was in the presence of testosterone compared to ethanol or estrogen (Figures 1C,D).

To further determine the effects of steroid hormones on QSM secretion and melanization, a dose-response analysis of both testosterone and estradiol was completed in conditioned media. Five concentrations of testosterone (0, 0.5, 2, 5, and 10 ng/mL) and estradiol (0, 100, 200, 400, and 10 ng/mL) were used to supplement conditioned L-DOPA media. These concentrations were chosen as a range of physiological concentrations. H99S was grown for 32 h in the various hormone concentrations and the absorbance at an optical density of 400 and 600 nm was measured (Supplementary Figures 1A,B). As the concentration of both testosterone and estradiol increased, melanization also increased in *C. neoformans* (*p* = 0.02), indicating that both hormones affected melanization in *C. neoformans*.

### Steroid Hormones Affected the Secretion of Pantothenic Acid

**FIGURE 2:**
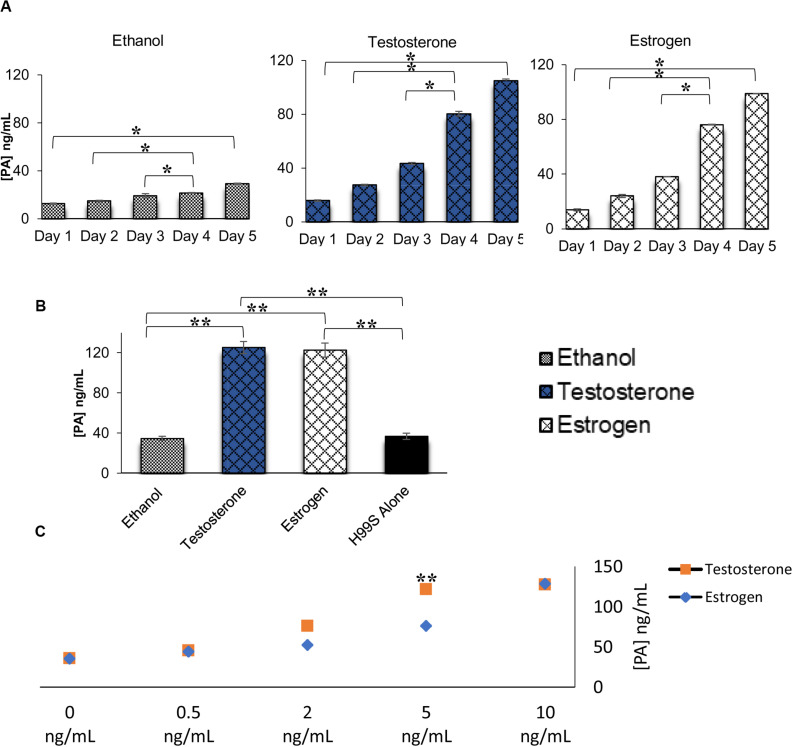
Pantothenic acid (PA) concentrations secreted from *C. neoformans* in various conditions using ELISA. **(A)** PA concentrations secreted from *C. neoformans* grown in steroid hormone-supplemented L-DOPA medium for 5 days. Error bars represent the standard deviation. Data is representative of three independent experiments. Statistical significance is represented as follows: *p < 0.01 comparing all days in each treatment, *p < 0.026 comparing Test to Estr over all days, using MANOVA with simple contrasts. **(B)** PA concentrations secreted from *C. neoformans* grown in steroid hormone-supplemented conditioned medium at the 32 h time point. Error bars represent the standard deviation. Data is representative of three independent experiments. Statistical significance is represented as follows: **p < 0.001, using MANOVA with simple contrasts. **(C)** PA concentrations secreted from *C. neoformans* while grown in various concentrations of testosterone and estradiol. The *X*-axis depicts the concentrations of testosterone and estradiol. Error bars represent the standard deviation. Data is representative of two independent experiments. **p < 0.001, using MANOVA with simple contrasts. Test, Testosterone; Estr, Estrogen (Estradiol); Eth, Ethanol.

ELISAs were completed on H99S samples to determine the concentration of PA secreted in various conditions. When PA secretion was compared between *C. neoformans* in steroid hormone treatments over 5 days of melanization, there was no difference between testosterone and estradiol, and both hormones affected PA secretion when compared to the ethanol control (Figure 2A). To determine if the presence of hormones affected fungal growth, which might explain the increased secretion of PA, we conducted a growth curve of H99S in the presence of steroid hormones. While there was similar growth in the presence of testosterone and estrogen, there was significantly increased growth in both hormones compared to growth in ethanol (Supplementary Figure 2), suggesting that steroid hormones were likely partly responsible for the increased PA secretion. There was significantly more PA present at day five compared to previous days in all hormones, as expected, due to the cell density increase (*p* = 0.01). ELISAs completed on conditioned media (Figure 2B) and steroid hormone dose-response samples (Figure 2C) also indicated no difference in PA secretion when comparing testosterone to estradiol (except for one concentration in the dose-response curves), suggesting that PA secretion was not causing the melanization difference observed during the initial experiments.

### qRT-PCR and RNA Sequencing Analysis Revealed Gene Expression Changes in *C. neoformans* When Grown in the Presence of Steroid Hormones

Due to the known effects of steroid hormones on gene regulation (Parker, 1988; Ing, 2005), we hypothesized that testosterone’s effects were at the transcriptional level. Since it was initially thought that the observed melanization difference between testosterone and estrogen could be due to a QSM, qRT-PCR was utilized to determine gene expression of genes identified from the literature, which were known to be either involved in *C. neoformans* melanization or were *C. neoformans* homologs of other known QSMs (Hornby et al., 2001; Eisenman et al., 2007 (Table 1). The testosterone- and estrogen-treated samples were compared to the ethanol-treated samples (the vehicle control). When *C. neoformans* was in the presence of testosterone, these genes were upregulated compared to when *C. neoformans* was in the presence of estradiol. Thus, to determine changes in the entire transcriptome, RNA sequencing was pursued.

**TABLE 1:** qRT-PCR of genes thought to be involved in the melanization of *C. neoformans* or homologs of quorum sensing molecules (QSMs) found in other organisms in the presence of testosterone compared to estradiol.

Locus	Putative Function	^1^Real-Time PCR (geometric mean)	Significance to melanization in *C. neoformans*
CNAG_03202	Adenylate Cyclase	27.34	*C. neoformans* homologs of the QSM, farnesol, pathway in *C. albicans* (Hornby et al., 2001)
CNAG_01672	RAS1	8.94	
CNAG_01889	Glutathione *S*-Transferase	7.87	
CNAG_03922	Hypothetical Protein	2.23	Genes found in microarray study, thought to be involved in melanization in *C. neoformans* (Lee et al., 2007; Eisenman et al., 2011)
CNAG_02153	TUP1	3.25	
CNAG_03465	LAC1	592.34	Gene responsible for the synthesis of melanin in *C. neoformans* (Eisenman et al., 2007)

*^1^Fold changes reflect the differences between testosterone versus estrogen after being normalized by the geometric mean of two different housekeeping genes (Vandesompele et al., 2002).*

RNA sequencing analysis comparing *C. neoformans* in the presence of steroid hormones was used to identify gene expression changes across the transcriptome during melanization. To determine what time point was best to extract RNA, qRT-PCR was conducted on levels of laccase mRNA in cells grown in L-DOPA media for 1, 2, or 3 days. Laccase mRNA was upregulated on day 1, but not on days 2 or 3 (data not shown). However, since *C. neoformans* cells visually started to melanize on day 3 (Figure 1A), it was decided to extract RNA from *C. neoformans* on day three. Then, cDNA libraries were prepared and sent for sequencing. For data analysis, the testosterone-treated samples were compared to both the estrogen-treated and ethanol-treated samples. These data revealed four genes that showed significant upregulation in *C. neoformans* in the presence of testosterone compared to estrogen (Table 2). CNAG_03340, a flavonol synthase, is a transmembrane protein that is part of the gibberellic biosynthesis pathway (FungiDB, 2019). Given this finding, other genes in the gibberellic biosynthesis pathway in the RNA sequencing data were identified, including four additional genes: cytochrome p450 monooxygenase pc-2 (CNAG_02841), cytochrome p450 (CNAG_05842 and CNAG_04029), and sphingolipid delta-4 desaturase (CNAG_00644) (Table 2). The gibberellic biosynthesis pathway produces GA, which typically increases growth rates in other organisms (Hedden et al., 2001; Tudzynski, 2005). This pathway has not been previously identified in *C. neoformans*.

**TABLE 2:** Significant gene expression of H99S (above CNAG_03465) and genes thought to be involved in a gibberellic acid biosynthesis pathway (below CNAG_13056) in the presence of testosterone on day three of melanization from RNA sequencing and qRT-PCR analysis.

Locus	Putative Function	RNA Seq. Fold Change	^2^Real-Time PCR Fold Change	Validated	Significant (RNAseq)
CNAG_03340	Flavonol Synthase	17.88	75.04	Yes	Yes
CNAG_05356	Hypothetical Protein	45.84	23.82	Yes	Yes
CNAG_03857	Hypothetical Protein	18.41	25.55	Yes	Yes
CNAG_13056	Hypothetical RNA (ncRNA)	88.47	57.31	Yes	Yes
CNAG_03465	Laccase	1.6	592.34	Yes	No
CNAG_02841	Cytochrome P450 Monooxygenase	1.59	11.68	Yes	No
CNAG_05842	Cytochrome P450	1.36	8.08	Yes	No
CNAG_04029	Cytochrome P450	3.19	19.54	Yes	No
CNAG_00644	Sphingolipid Delta-4 Desaturase	1.34	6.42	Yes	No

*^2^Fold changes reflect the differences between testosterone versus estrogen after being normalized by the geometric mean of two different housekeeping genes (Vandesompele et al., 2002). The validated column indicates whether or not the RT-PCR fold change matched the RNAseq fold change.*

### Steroid Hormones Affected the Secretion of Gibberellic Acid

**FIGURE 3:**
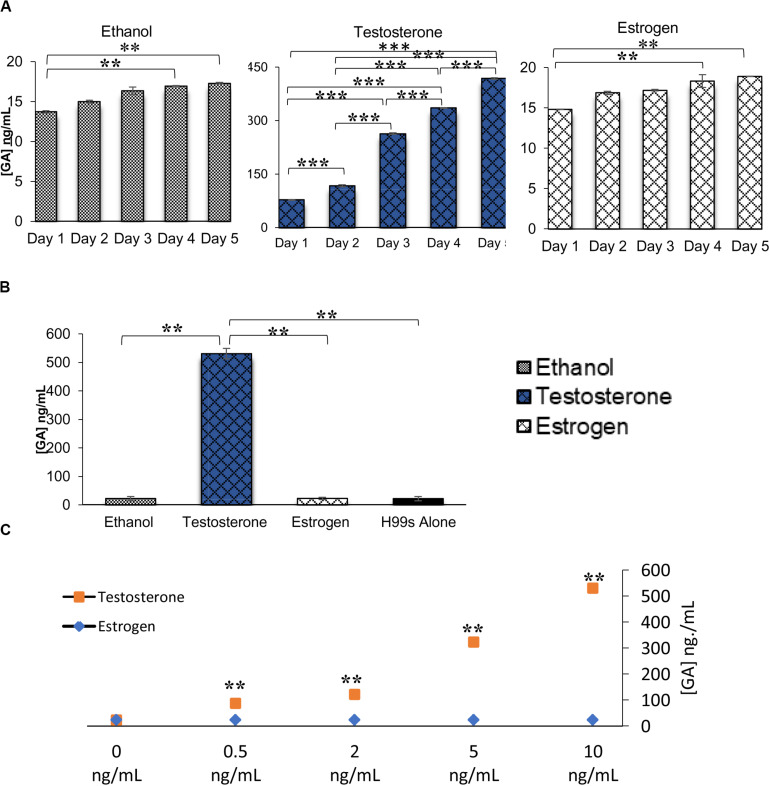
Gibberellic acid (GA) concentrations secreted from *C. neoformans* in various conditions measured via ELISA. **(A)** GA concentrations secreted from *C. neoformans* grown in steroid hormone-supplemented L-DOPA medium for 5 days. Error bars represent the standard deviation. Data is representative of three independent experiments. Statistical significance is represented as follows: in Ethanol treatment **p = 0.0167 comparing Day 1 and Day 4 and **p = 0.0089 comparing Day 1 to Day 5, in Testosterone treatment all days are different ***p < 0.001, in Estrogen treatment **p = 0.01 comparing Day 1 and Day 4 and **p = 0.0037 comparing Day 1 to Day 5, using MANOVA with simple contrasts. **(B)** GA concentrations secreted from *C. neoformans* grown in steroid hormone-supplemented conditioned medium at the 32 h time point. Error bars represent the standard deviation. Data is representative of three independent experiments. Statistical significance is represented as follows: **p < 0.001 using Tukey–Kramer HSD. **(C)** GA concentrations secreted from *C. neoformans* while grown in various concentrations of testosterone or estrogen (estradiol). The *X*-axis depicts the concentrations of testosterone. Error bars represent the standard deviation. Data is representative of two independent experiments. **p < 0.001, using MANOVA with simple contrasts.

RNA sequencing analysis revealed multiple genes that were part of a gibberellic biosynthesis pathway that were upregulated during melanization of *C. neoformans* in the presence of testosterone. ELISAs were completed on multiple H99S samples to determine if GA was secreted by *C. neoformans* and at what concentration. Basal levels of GA secretion by *C. neoformans* were first determined in L-DOPA media without steroid hormones over a 3-day period. *C. neoformans* secreted similar levels of GA per day: 13 ng/mL on day 1, 15 ng/mL on day 2, and 16 ng/mL GA on day 3. These data were similar to those measured in the presence of ethanol during a 5-day melanization experiment (Figure 3A) and a conditioned media experiment (Figure 3B). ELISAs of these experiments showed a significant increase in GA secretion when *C. neoformans* was in the presence of testosterone compared to estradiol (*p* < 0.05). When comparing ELISAs completed on the dose-response of both testosterone and estradiol, the difference in GA secretion was striking (Figure 3C). When *C. neoformans* was in the presence of testosterone, there was a dose-dependent increase in GA secretion as testosterone concentration increased. These data were similar to the PA ELISA completed on the testosterone dose-response (Figure 2C). However, when *C. neoformans* was in the presence of estradiol there was no change in GA secretion when estradiol concentration varied. This led to the hypothesis that testosterone was regulating GA secretion, and this secretion could be causing the difference in melanization observed when hormones were present.

**FIGURE 4:**
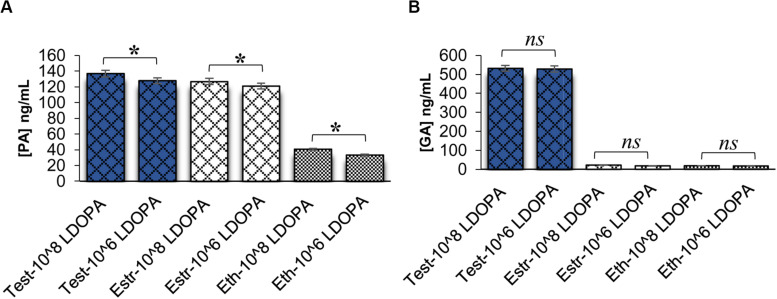
Pantothenic acid (PA) and gibberellic acid (GA) concentrations secreted from *C. neoformans* at different cell densities using ELISA. **(A)** Comparison of PA concentrations secreted from *C. neoformans* grown in L-DOPA medium supplemented with steroid hormones at two cell densities. Error bars represent the standard error of the mean. Data is representative of three independent experiments. Statistical significance is represented as follows: *p < 0.001 using Tukey–Kramer HSD. **(B)** Comparison of GA concentrations secreted from *C. neoformans* grown in L-DOPA medium supplemented with steroid hormones at two cell densities. Error bars represent the standard error of the mean. Data is representative of three independent experiments. Statistical significance is represented as follows: *p* > 0.05 using Tukey–Kramer HSD, ns, not significant. Test, testosterone; Estr, Estrogen (Estradiol); Eth, Ethanol.

We further hypothesized that GA could be acting as a QSM, similar to PA. However, when this hypothesis was tested, it was found that GA was not a QSM. Figure 4A reveals ELISAs completed on *C. neoformans* at different cell densities in the presence of steroid hormones. As cell density increased, PA concentrations also increased (*p* < 0.001), as expected for a QSM. However, GA concentrations remained the same between cell densities (*p* > 0.05) (Figure 4B); revealing that GA was not a QSM, but instead could potentially function as a growth hormone whose production was regulated by testosterone; thus, affecting melanization.

### GA Affected Melanization and Gene Expression of *C. neoformans*

**FIGURE 5:**
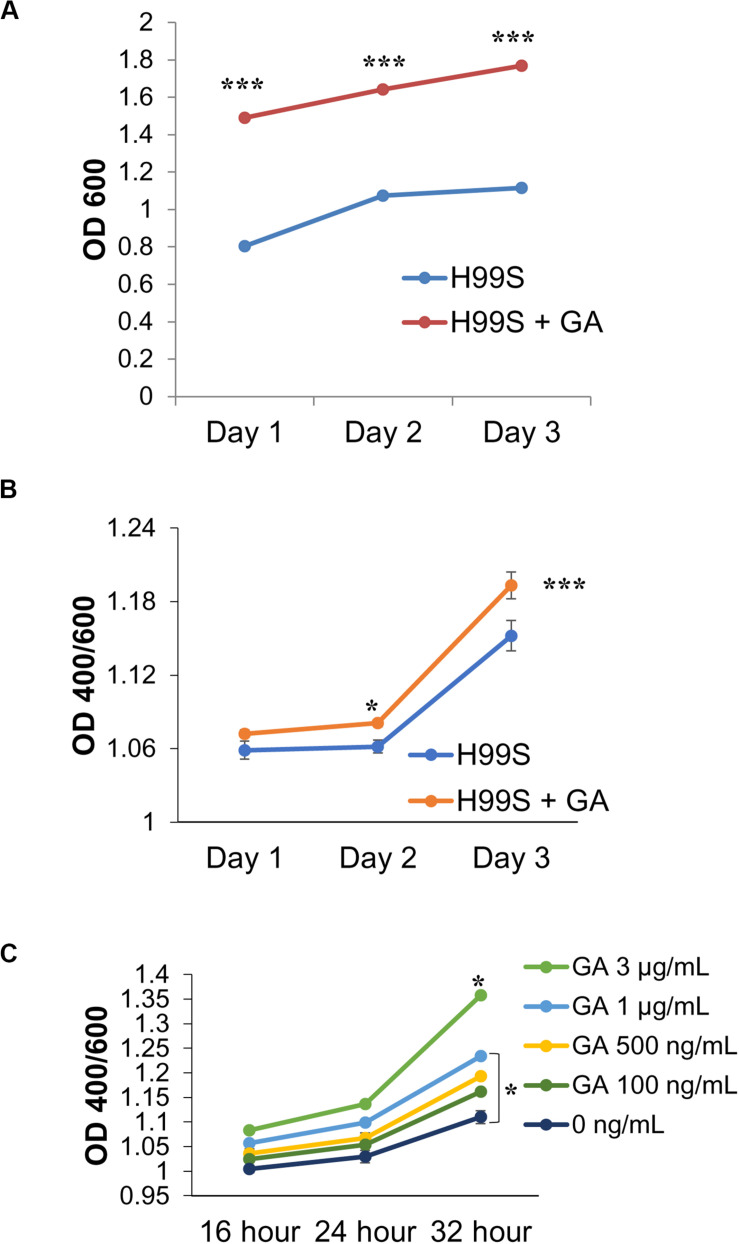
Gibberellic acid (GA) effects on *C. neoformans* growth and melanin production. **(A)** A comparison of H99S growth with and without exogenous GA (3 μg/mL). Error bars represent the standard deviation. Data is representative of three independent experiments. Statistical significance is represented as follows: ***p < 0.0001 for all days, using MANOVA with simple contrasts. Absorbances were measured at an optical density of 400 and 600 nm. **(B)** A comparison of H99S melanization with and without exogenous GA (3 μg/mL). Error bars represent the standard deviation. Data is representative of three independent experiments. Statistical significance is represented as follows: *p = 0.012 comparing Day 2 and ***p < 0.0001 comparing Day 3, using MANOVA with simple contrasts. Absorbances were measured at an optical density of 400 and 600 nm. **(C)** Dose-response of exogenous GA at various concentrations in conditioned media. Error bars represent the standard deviation. Data is representative of three independent experiments. Statistical significance is represented as follows: *p < 0.02 comparing 3 μg/mL to all other concentrations and *p = 0.01 comparing 1 μg/mL to 0 ng/mL using linear regression.

To determine if GA was acting as a growth hormone and to understand the effects GA had on *C. neoformans*, exogenous GA was added to H99S samples without the presence of steroid hormones. Growth experiments showed that addition of GA significantly increased the optical density, suggesting GA was acting as a growth hormone (*p* < 0.0001 for all days, Figure 5A). Melanization experiments revealed that when *C. neoformans* was in the presence of exogenous GA, there was an increase in melanization compared to H99S alone (*p* < 0.0001, Figure 5B). A dose-response using various concentrations of GA was also completed (Figure 5C) showing that as GA concentration increased, melanization of *C. neoformans* also increased (*p* < 0.02). This indicated that GA had an effect on melanization, likely as a growth hormone, and when secreted, resulted in increased cell density.

The GA pathway in *C. neoformans* has yet to be discussed in the literature. However, this growth hormone is found in other fungi, such as *Gibberella fujikuroi* (McInnes et al., 1977), making it more likely to be found in *C. neoformans* as well. Supplementary Figure 3 shows the GA pathway in *Gibberella fujikuroi* and where flavonol synthase is located in the pathway. This gene was identified in the RNA sequencing data and was significantly upregulated when *C. neoformans* was in the presence of testosterone.

### Phenotypic Testing of KO Strains of Genes Involved in the GA Pathway Revealed a Decrease in Melanization in the Presence of Testosterone

**FIGURE 6:**
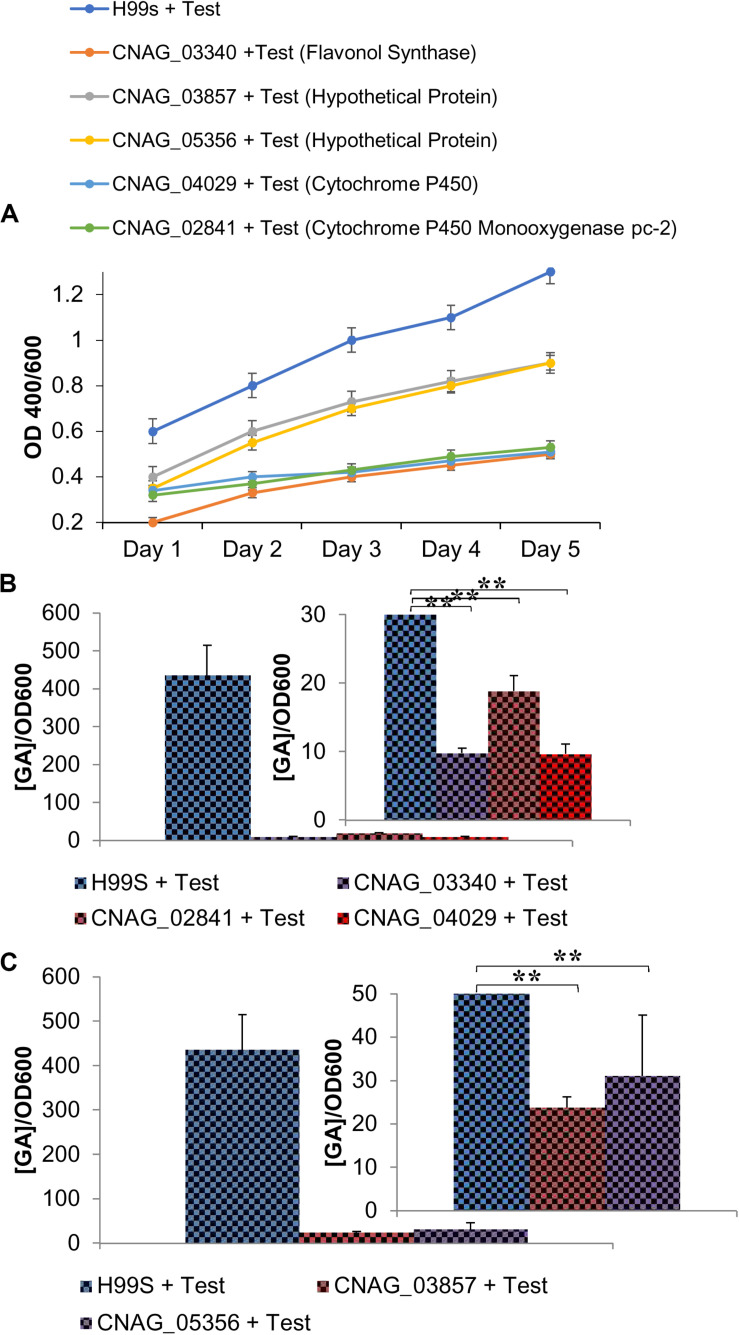
Phenotypic changes when genes involved in the gibberellic acid (GA) pathway are knocked out (KO) of *C. neoformans*. **(A)** Melanization of *C. neoformans* KO strains identified in the RNAseq data. Absorbances were measured at an optical density of 400 and 600 nm. Error bars represent the standard deviation. Data is representative of three independent experiments. Statistical significance is represented as follows: ***p < 0.0001 comparing H99S + Test (10 ng/mL) with all other strains, ***p < 0.0001 comparing strains CNAG_03857 to CNAG_04029, CNAG_5356 to CNAG_04029, CNAG_04029 to CNAG_02841, CNAG_04029 to CNAG_03340, and CNAG_02841 to CNAG_03340 using linear regression. **(B)** GA concentrations (ng/mL) secreted from KO strains at 32 h of melanization normalized to an optical density of 600 nm. Error bars represent the standard deviation. Data is representative of three independent experiments. Statistical significance is represented as follows: **p < 0.004 using Wilcoxon Rank Sums. **(C)** Gibberellic acid (GA) concentrations (ng/mL) secreted from KO strains of two hypothetical proteins identified in the RNA sequencing data that were upregulated when *C. neoformans* was in the presence of testosterone at 32 h of melanization normalized to an optical density of 600 nm. Error bars represent the standard deviation. Data is representative of three independent experiments. Statistical significance is represented as follows: **p < 0.004 using Wilcoxon Rank Sums. Test, Testosterone; Estr, Estrogen (Estradiol); Eth, Ethanol.

To determine if the GA biosynthesis pathway was involved in melanization, KO strains of genes identified in the RNA sequencing analysis, including various genes involved in the GA biosynthesis pathway, were tested for amounts of melanization in L-DOPA media plus testosterone (over 5 days) compared to H99S plus testosterone. All of the KO strains tested in liquid medium showed less melanization over 5 days (Figure 6A) when compared to H99S plus testosterone, and the KO strains of genes involved in the GA biosynthesis pathway showed the least amount of melanization. If *C. neoformans* was producing GA it was likely that these genes (cytochrome p450 monooxygenase pc-2, flavonol synthase, the hypothetical proteins, and cytochrome p450) all played a role in *C. neoformans* melanization in testosterone. A growth curve of the KO strains was also completed to compare growth rates in the KO strains to the H99S wildtype strain in L-DOPA media (Supplementary Figure 4). All of the KO strains grew significantly slower than the wildtype strain, H99S, regardless of the addition of exogenous hormone, which could be confounding the results. However, at the 5-day time point, the color of the KO strains were all a very light brown, indicating that these strains melanized, just at reduced levels (data not shown).

To determine the concentration of GA during melanization of the KO strains of genes in the GA biosynthesis pathway, ELISAs were conducted to determine the amount of GA secreted at 32 h in conditioned media (Figure 6B). GA concentrations varied widely when the KO strains were compared to H99S plus testosterone, so the concentrations were normalized by the optical density at 600 nm to control for cell density. In the KO strains, the normalized concentrations ranged from 10–20 ng/mL compared to 436 ng/mL in H99S plus testosterone. Additionally, the KO strains of the two hypothetical proteins that were identified in the RNA sequencing were also tested for GA secretion during melanization at 32 h (Figure 6C). As observed for the KO strains of genes in the GA biosynthesis pathway, these strains also showed reduced normalized secretion of GA compared to H99S plus testosterone (23–31 ng/mL compared to 436 ng/mL in H99S plus testosterone). These data suggested that both the hypothetical proteins and the genes in the GA biosynthesis pathway affected GA production.

### Melanization of *C. neoformans* Was Decreased in Conditioned Media Supplemented With Steroid Hormones and Exogenous Inhibitors

**FIGURE 7:**
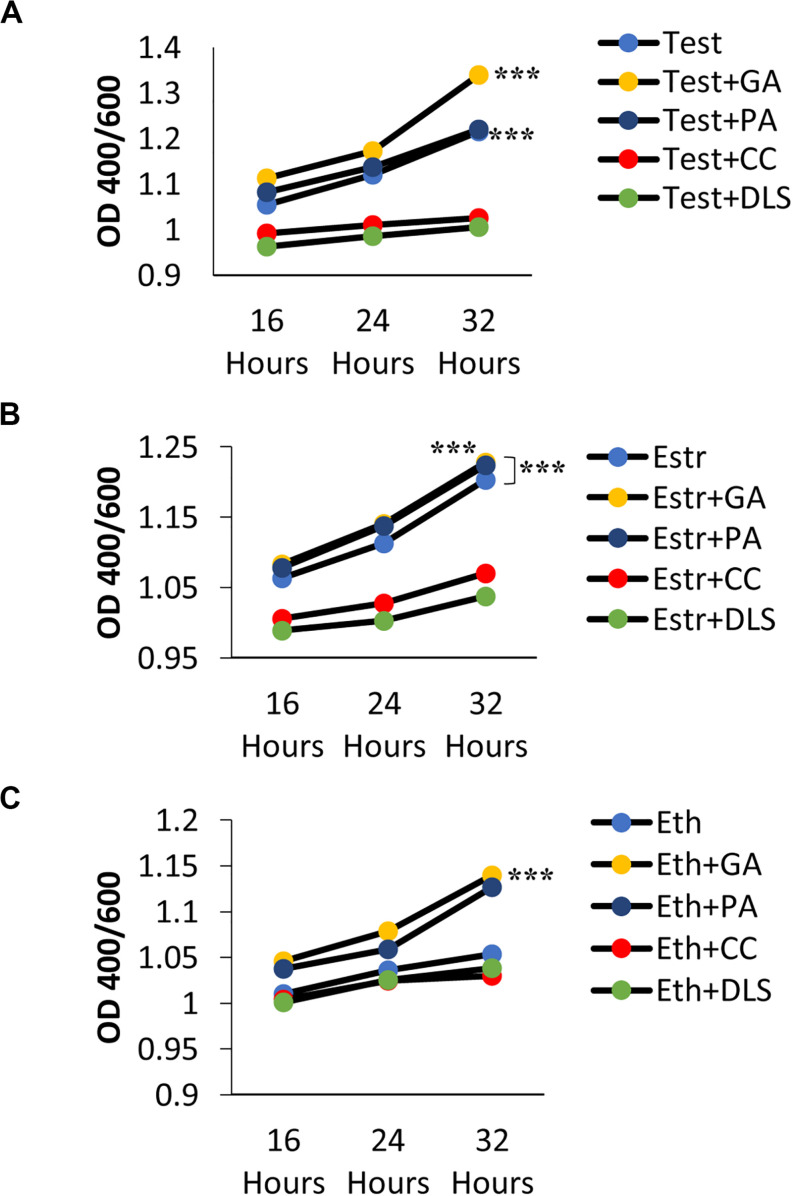
Melanization of *C. neoformans* grown in the presence of various additives/inhibitors and steroid hormones. **(A)** Testosterone, **(B)** Estrogen, **(C)** Ethanol. Absorbances were measured at an optical density of 400 and 600 nm. Error bars represent the standard deviation. Data is representative of three independent experiments. Statistical significance is represented as follows: ***p < 0.0001 comparing Test alone and Test + GA to all other treatments. ***p < 0.0001 comparing Estr + GA to Estr alone and Estr + PA to all other treatments. ****p < 0.0001 comparing Eth + GA to all other treatments using linear regression. Test, Testosterone; GA, Gibberellic Acid; PA, Pantothenic Acid; CC, Chlormequat Chloride; DS/DLS, DL-Serine.

To verify that the GA biosynthesis pathway was involved in melanization, another conditioned media experiment with exogenous inhibitors of GA and PA was conducted (Figures 7A–C). All treatments of H99S contained GA, chlormequat chloride (a GA inhibitor), PA, or DL-Serine (a PA inhibitor) plus the presence of steroid hormones. When exogenous GA and testosterone were added to *C. neoformans*, there was a significant increase in melanization compared to *C. neoformans* in the presence of exogenous PA and testosterone. Chlormequat chloride and DL-Serine are known to inhibit 50% of GA and PA production, respectively, in other organisms (Rademacher, 1992). This experiment demonstrated a significant decrease in melanization when chlormequat chloride or DL-Serine was present, suggesting that *C. neoformans* was producing both GA and PA molecules, and their production was being inhibited; thereby, decreasing melanization in *C. neoformans*.

### Theoretical Model of How Testosterone Regulates GA Production

We hypothesized that due to the GA concentration only being increased in the presence of testosterone compared to estradiol (Figure 3C) and the fact that GA had an effect on melanization (Figure 5B), testosterone was regulating GA production, which affected melanization, resulting in the melanization differences we observed when *C. neoformans* was in the presence of steroid hormones. RNA sequencing data revealed a hypothetical lncRNA (>200 nt) that was upregulated 88-fold when *C. neoformans* was in the presence of testosterone. When the lncRNA sequence was used to search for sequence homology using the BLAST algorithm (NCBI), multiple genes, including another gene identified in the RNAseq data (CNAG_05356), with transmembrane domains were identified (Table 3). CNAG_05356 is a hypothetical protein that shows decreased melanization and decreased GA secretion when knocked out, as shown in Figures 6A,C, indicating this gene is involved in GA secretion in *C. neoformans*.

**TABLE 3:** Genes identified when the hypothetical lncRNA (486 bp) identified in the RNAseq data is used to search for sequence homology using the BLAST algorithm from NCBI and their percentage of coverage.

Gene Identified by Blast	Putative Function	Percent Covered	# of BPs covered	# of Transmembrane domains
CNAG_04597	Hypothetical Protein	39%	193	11 (Throughout)
CNAG_05356	Hypothetical Protein	24%	118	1 (at beginning)
CNAG_04331	Hypothetical Protein	22%	107	0
CNAG_00229	Hypothetical Protein	22%	106	0

**FIGURE 8:**
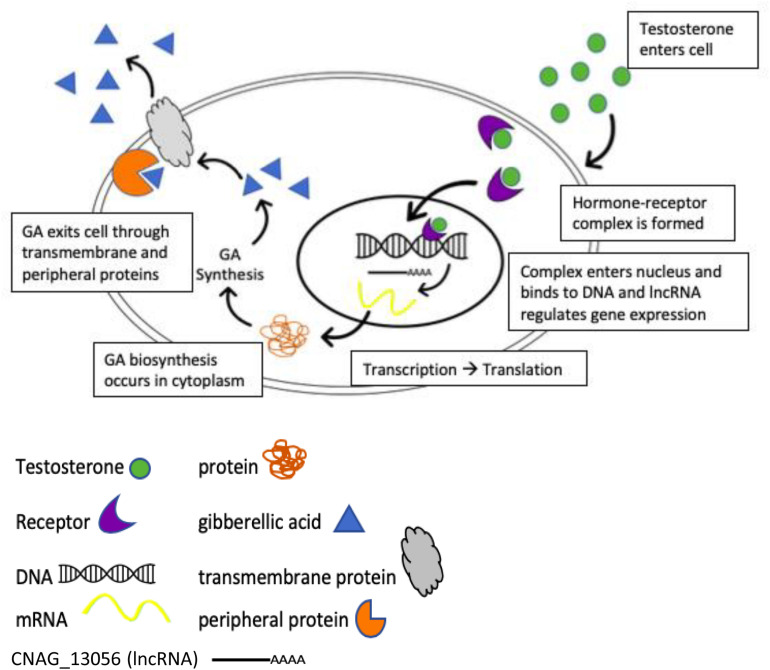
Potential model for the regulation of gibberellic acid production when *C. neoformans* is in the presence of testosterone.

Assuming testosterone enters *C. neoformans* cells, we hypothesize that testosterone upregulates transcription of GA and the hypothetical lncRNA is activated by testosterone to regulate transcription or translation of genes that encode proteins containing transmembrane domains that were identified in the BLAST search, and the RNA sequencing data, which then play a role in the transport and release of GA from the cell (Figure 8). Once GA is released from the cell, it acts as a growth hormone to increase proliferation of *C. neoformans*, which results in increased fungal growth and melanization, once a high enough cell density has been reached.

### Biological Relevance

**FIGURE 9:**
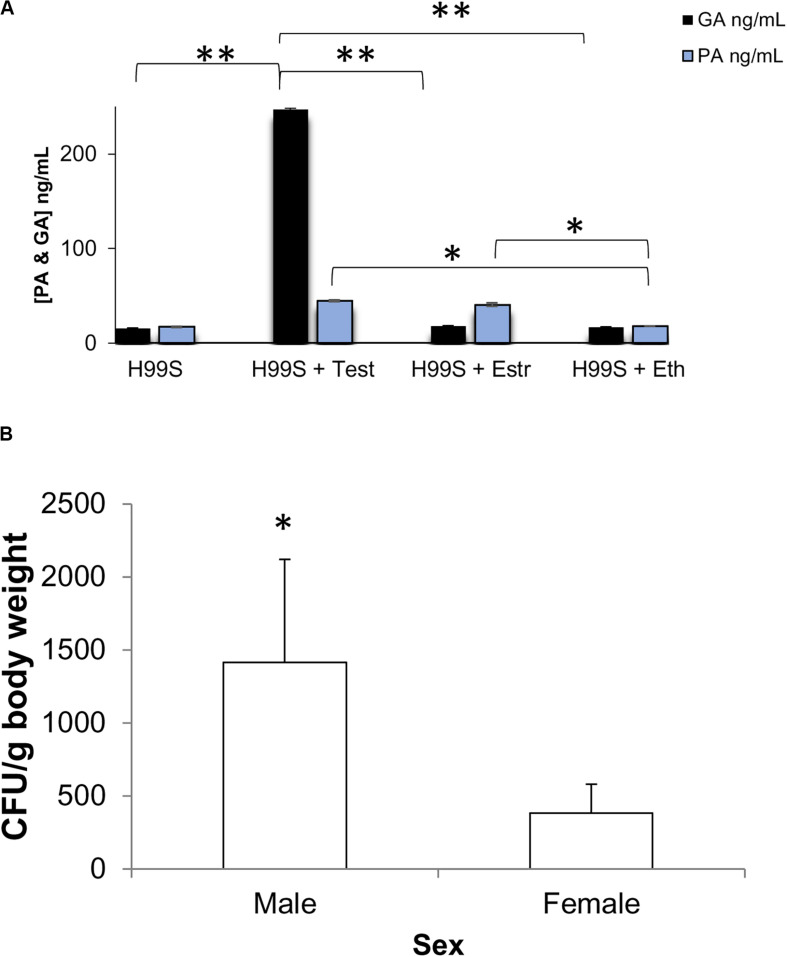
Biological relevance **(A)** Gibberellic acid (GA) and Pantothenic acid (PA) concentrations after incubation at 37°C + 5% CO_2_. Error bars represent the standard deviation. Data is representative of three independent experiments. ^∗∗^*p* < 0.0001 Test vs. all other conditions; ^∗^*p* < 0.05 Est and Test vs. Eth and media alone; ANOVA with Tukey’s HSD. **(B)** Combined organ fungal burden per gram body weight at day 7 post-infection in male and female mice infected with H99S. Error bars represent the standard error of the mean. Data are representative of one experiment, *N* = 6 mice/sex. ^∗^*p* = 0.0345, Wilcoxon Ranks Sums. Test, Testosterone; Estr, Estrogen (Estradiol); Eth, Ethanol.

As GA production is required for the fungus *G. fujikuroi* to cause bakanae disease in rice (Studt et al., 2013), GA production may also contribute to pathogenesis in *C. neoformans*. To determine if GA was produced in other conditions than melanizing conditions, we incubated H99S in the presence of steroid hormones and 5% CO_2_ to mimic the environment of the host lung and then measured the concentration of GA and PA in the supernatant the next day. There was significantly more GA secreted from *C. neoformans* incubated in the presence of testosterone (*p* < 0.0001) than any other condition, while there were significantly higher concentrations of PA secreted from *C. neoformans* incubated in the presence of estradiol and testosterone than in ethanol or media alone (*p* < 0.05, Figure 9A).

We next infected male and female BALB/c mice with H99S to determine if there was a difference in fungal burden *in vivo* in the presence of physiological levels of hormones. While there was no difference in survival or fungal burden in each individual organ (spleen, lungs, or brain) between males and females, at 7 days post-infection, males had a significantly higher overall fungal burden than females when the fungal burden from all tested organs was summed (*p* = 0.0345, Figure 9B).

## Discussion

The sex bias observed during *C. neoformans* infections has been a topic of debate as early as the 1960s (Guess et al., 2018). Although research on the effects of steroid hormones on *C. neoformans* melanin production is fairly new, this phenomenon has also been documented in animals (Fargallo et al., 2007). By characterizing the effects of testosterone and estrogen on *C. neoformans* virulence factors, we hoped to unearth the cause of this difference.

Initial data (Figure 1) suggested that melanization differed in the presence of steroid hormones. A variety of experiments were conducted to validate this, including a modified conditioned media experiment (Eisenman et al., 2011) (Figures 1C,D). This experiment suggested that when “old” *C. neoformans* culture media with L-DOPA was added to a new culture, melanization occurred at an increased rate. This was similar to the findings of Eisenman et al. (2011), where the authors let *C. neoformans* grow for 7 days in minimal medium and then added L-DOPA. With the late addition of L-DOPA to the old culture, the authors showed an increase in melanization compared to a *C. neoformans* culture that was initially grown in L-DOPA. Although Eisenman et al. (2011) did not test the effects of steroid hormones on melanization, both studies suggest that *C. neoformans* was secreting a molecule(s) that increased melanization, further suggesting a quorum sensing-like mechanism. Additionally, our conditioned media experiment with steroid hormones revealed that when *C. neoformans* melanized in the presence of testosterone, melanization was increased compared to *C. neoformans* melanizing in the presence of estradiol. These data suggested that when *C. neoformans* melanized in the presence of testosterone, there was an increase in the secretion of these molecules.

To better understand steroid hormone effects on melanization in *C. neoformans*, a dose-response experiment using both testosterone and estradiol was completed (Supplementary Figures 1A,B). By manipulating the concentration of hormone present in the *C. neoformans* culture we determined if the amount of melanin produced could be altered. Interestingly, this study found that as the concentration of either hormone increased, the amount of melanin also increased.

Microarray data by Eisenman et al. (2011) identify genes that are upregulated when *C. neoformans* is melanized. Those genes were also shown to be upregulated by qRT-PCR (Table 1) when *C. neoformans* melanized in the presence of testosterone compared to estrogen. However, results of RNA sequencing indicated several other genes were upregulated when *C. neoformans* melanized in testosterone (Table 2). Interestingly, laccase, a gene responsible for synthesizing melanization in *C. neoformans* (Gómez and Nosanchuk, 2003), was upregulated when *C. neoformans* was in the presence of testosterone compared to estradiol nearly 600-fold in the qRT-PCR screen (Table 1), but only 1.6-fold in the RNA sequencing data (Table 2). We hypothesize that this drastic fold change difference could be due to the use of different primers for laccase in the two experiments and the many splice regions located within the gene (Dr. Rebecca Seipelt-Thiemann, personal communication). Thus, as expected, laccase likely contributed to the melanization difference observed when *C. neoformans* was in the presence of steroid hormones, after a molecule that has yet to be described in *C. neoformans*, GA, was upregulated by the presence of testosterone. The RNA sequencing data showed multiple genes that were upregulated, including flavonol synthase, which plays a major role in the GA biosynthesis pathway. Thus, we hypothesized that the increase in expression of these genes in the presence of testosterone contributed to the observed difference in melanization.

GA is secreted by many other fungi and is thought to aid survival in harsh environments and increase pathogenesis (Salazar-cerezo et al., 2018). It is also a growth hormone most commonly found in plants (Hedden et al., 2001). Due to its effects in other organisms we hypothesized that like PA, GA could also be acting as a QSM in *C. neoformans* and affecting melanization. However, this hypothesis was found to be incorrect. Instead, we next hypothesized that testosterone was regulating the production of GA, which was in turn affecting melanization by acting as a growth hormone to increase cell density. Indeed, when exogenous GA was added to H99S, increased growth was observed (Figure 5A), suggesting GA acted as a growth hormone in *C. neoformans*.

While there were small differences in GA production in the presence of estrogen and ethanol (Figure 3A) and it is unlikely these are biologically relevant, because it is unknown how much of an effect even a small amount of GA could have on fungal growth and melanization, it is possible that even small differences in GA levels would be biologically relevant. For example, in plants, concentrations as low as 10^–8^ M result in increased growth in the diameter of leaves (Chandler and Robertson, 1999). However, the 24-fold increase in GA production when *C. neoformans* was in the presence of testosterone compared to estradiol (Figure 3C) was clearly biologically relevant. *C. neoformans* grown in the presence of estradiol showed no change in GA production, suggesting that GA was regulated by testosterone. In addition, when we added different concentrations of GA, we saw a dose-dependent increase in melanization, and melanization experiments conducted in KO strains of genes in the GA biosynthetic pathway and in H99S in the presence of a GA inhibitor showed inhibition of melanization, suggesting that the GA biosynthesis pathway was involved in melanization. Since we were not specifically determining the function of each individual gene in the GA biosynthesis pathway, but rather that the pathway itself was contributing to melanization in *C. neoformans*, we did not test melanization of the reconstituted strains of these genes. To the best of our knowledge, this is the first report of the production of GA and its effects on melanization in *C. neoformans*.

The adaptation for increased melanization in the presence of testosterone could have possibly evolved from hormones secreted into the environment. Steroid hormones are naturally released into the extracellular environment by both humans and animals (Shore and Shemesh, 2003) and are stable insoluble peptides that are not broken down when excreted (Shore and Shemesh, 2003). The amount of hormone secreted depends on a variety of factors (i.e., diet, pregnancy, pharmaceutical consumption, and the type of animal) (Shore and Shemesh, 2003). Regardless of the source, these excreted hormones may have potentially aided the adaptation of *C. neoformans* to testosterone, resulting in the increased secretion of GA in the presence of testosterone, which could have downstream consequences. Increased production of GA would rapidly increase fungal cell numbers in the male host, leading to a more rapid melanization process *in vivo*. As melanization is known to inhibit phagocytosis (Casadevall et al., 2000) and protect the cell from reactive oxygen species (Wang et al., 1995), this could hamper the male immune response to the infection leading to increased fungal burden and disease in males, which could, in part, explain the sex-bias observed in cryptococcosis infections.

To test whether GA contributed to pathogenesis in *C. neoformans*, we incubated H99S cells in conditions that mimicked the environment of the host lung and then determined if PA or GA were secreted. Interestingly, there was significantly higher levels of PA secreted in the presence of both estradiol and testosterone compared to ethanol or media alone, suggesting that PA secretion was equally affected by both steroid hormones, but was not necessarily secreted in conditions that mimic the host lung. While there were significantly higher amounts of GA secreted in the presence of testosterone, there was no difference between any of the other conditions, suggesting that GA production was not turned on in conditions that mimic the environment of the host lung, so GA may not be required for pathogenesis. Alternatively, perhaps GA production is only turned on in the presence of testosterone. If this is the case, then increased production of GA in the presence of testosterone would result in increased proliferation of *C. neoformans*, increased melanization, and result in increased overall fungal burden at day 7 post-infection in male mice, as we observed (Figure 9B). A previous mouse experiment we conducted using H99W (Janbon et al., 2014) in male and female BALB/c mice also showed that male mice had higher spleen and brain fungal burden during a chronic infection (day 39 post-infection) (McClelland et al., 2013). However, even with the increased fungal burden in males, this did not translate into significant differences in survival between males and females. Nonetheless, since the survival experiment was only done once and so little is known about how GA affects fungal biology, these experiments and hypotheses will need to be tested further in the future.

Long non-coding RNAs (lncRNA) have been known to regulate gene expression in a variety of models. The lncRNA, Malat1, controls the expression of genes involved in nuclear processes and synaptogenesis (Bernard et al., 2010), while the expression of the lncRNA PCAT-1 causes a homologous recombination deficiency and the suppression of the tumor suppressor BRCA2, which has been linked to cancer (Prensner et al., 2014). Given the role that lncRNAs play in regulating transcription and translation, we hypothesize that CNAG_13056, a lncRNA that was significantly upregulated in the presence of testosterone (Table 1), regulates the expression of transmembrane proteins that are involved in the secretion of GA. A potential model based on the RNAseq data illustrates how testosterone may regulate secretion of GA via this lncRNA (Figure 8). Further studies are required to definitively test this model.

## Conclusion

We have identified a molecule in *C. neoformans*, GA, whose production was increased in the presence of testosterone and caused significantly increased fungal growth and melanization, which may contribute to increased disease in males. As melanin production increases virulence and contributes to antifungal resistance in *C. neoformans* (Rosas et al., 2000), disrupting the PA and GA pathways could cause a significant reduction in both growth and melanization. Thus, drugs found to inhibit either or both pathways could be potential targets for future research.

## Data Availability Statement

The datasets presented in this study can be found in online repositories. The names of the repository/repositories and accession number(s) can be found at: https://www.ncbi.nlm.nih.gov/, SAMN12877552. Transcriptome sequences of *C. neoformans* in the presence of steroid hormones can be accessed through NCBI’s Sequence Read Archive (SRA) (https://www.ncbi.nlm.nih.gov/sra) using the accession number SAMN12877552.

## Ethics Statement

All animal use complied with the standards described in the NIH Guide for the Care and Use of Laboratory Animals, The US Animal Welfare Act, PHS Policy on Humane Care and Use of Laboratory Animals and Middle Tennessee State University Institutional Animal Care and Use Committee guidelines. The protocol was approved by the Institutional Animal Care and Use Committee at Middle Tennessee State University (protocol #18-3004). Experiments were not randomized or blinded and were done once. For euthanasia, carbon dioxide overdose was used.

## Author Contributions

TG performed the initial melanization screen on L-DOPA plates and helped to writing the manuscript. JT performed and analyzed all remaining experiments and wrote the manuscript. EM designed all the experiments, analyzed the data, and wrote the manuscript. All authors contributed to the article and approved the submitted version.

## Conflict of Interest

The authors declare that the research was conducted in the absence of any commercial or financial relationships that could be construed as a potential conflict of interest.
