# Supplementary material for: The Role of Testosterone and Gibberellic Acid in the Melanization of Cryptococcus neoformans
Source: Front Microbiol. 2020 Aug 13;11:1921. doi: 10.3389/fmicb.2020.01921 (PMC7456850; doi:10.3389/fmicb.2020.01921)
Supplement: Supplementary file 1 [file Table_1.docx]

Supplementary Material

# Supplementary Data

## QSM Secretion Varied During Melanization of *C. neoformans* When Grown in Steroid Hormone-Supplemented Conditioned Medium

To further determine the effects of steroid hormones on QSM secretion and melanization, a dose-response analysis of both testosterone and estradiol was completed in conditioned media. Five concentrations of testosterone (0 ng/mL, 0.5 ng/mL, 2 ng/mL, 5 ng/mL, and 10 ng/mL) and estradiol (0 pg/mL, 100 pg/mL, 200 pg/mL, 400 pg/mL, and 10 ng/mL) were used to supplement conditioned L-DOPA media. These concentrations were chosen as a range of physiological concentrations. H99S was grown for 32 h in the various hormone concentrations and the absorbance at an optical density of 400 nm and 600 nm was measured (Supplementary Figure 1A & B). As the concentration of both testosterone and estradiol increased, melanization also increased in *C. neoformans* (*p* = 0.02), indicating that both hormones affected melanization in *C. neoformans*.

## Growth Curve of H99S in Steroid Hormone-Supplemented L-DOPA Medium

To determine if the presence of hormones affected fungal growth, which might explain the increased secretion of PA, we conducted a growth curve of H99S in the presence of steroid hormones. While there was similar growth in the presence of testosterone and estrogen, there was significantly increased growth in both hormones compared to growth in ethanol (Supplementary Figure 2), suggesting that steroid hormones were likely partly responsible for the increased PA secretion.

## Flavonol synthase and the GA pathway

The GA pathway in *C. neoformans* has yet to be discussed in the literature. However, this growth hormone is found in other fungi, such as *Gibberella fujikuroi* (McInnes et al. 1977), making it more likely to be found in *C. neoformans* as well. Supplementary Figure 3 shows the GA pathway in *Gibberella fujikuroi* and where flavonol synthase is located in the pathway. This gene was identified in the RNAseq data and was significantly upregulated when *C. neoformans* was in the presence of testosterone.

## Growth Curve of H99S vs. Knockout Strains (KO) in Steroid Hormone-Supplemented L-DOPA Medium

A growth curve of the KO strains was also completed to compare growth rates in the KO strains to the H99S wildtype strain in L-DOPA media (Supplementary Figure 4). All of the KO strains grew significantly slower than the wildtype strain, H99S, regardless of the addition of exogenous hormone.

## Supplementary Figures

B.

A.


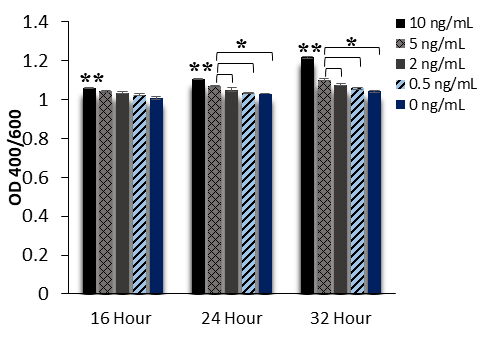


*****

*****

*****

*****

Supplementary Figure 1. A: Melanization of *C. neoformans* in conditioned media supplemented with various concentrations of testosterone. Absorbances were measured at an optical density of 400 nm and 600 nm. Error bars represent the standard deviation. Data is representative of three independent experiments. Statistical significance is represented as follows: ***p* < 0.0001 comparing 10 ng/mL to all concentrations, **p* = 0.0147 comparing 5 ng/mL to 2 ng/mL, **p* = 0.00013 comparing 5 ng/mL to 0.5 ng/mL and **p* = 0.0007 comparing 5 ng/mL to 0 ng/mL, using linear regression analysis. B: Melanization of *C. neoformans* in conditioned media supplemented with various concentrations of estradiol. Absorbances were measured at an optical density of 400 nm and 600 nm. Error bars represent the standard deviation. Data is representative of three independent experiments. Statistical significance is represented as follows: ***p* < 0.0001 comparing 400 pg/mL to all concentrations except 10 ng/mL, using linear regression analysis. Abbreviations: Test; Testosterone, Estr; Estrogen (Estradiol), Eth; Ethanol.


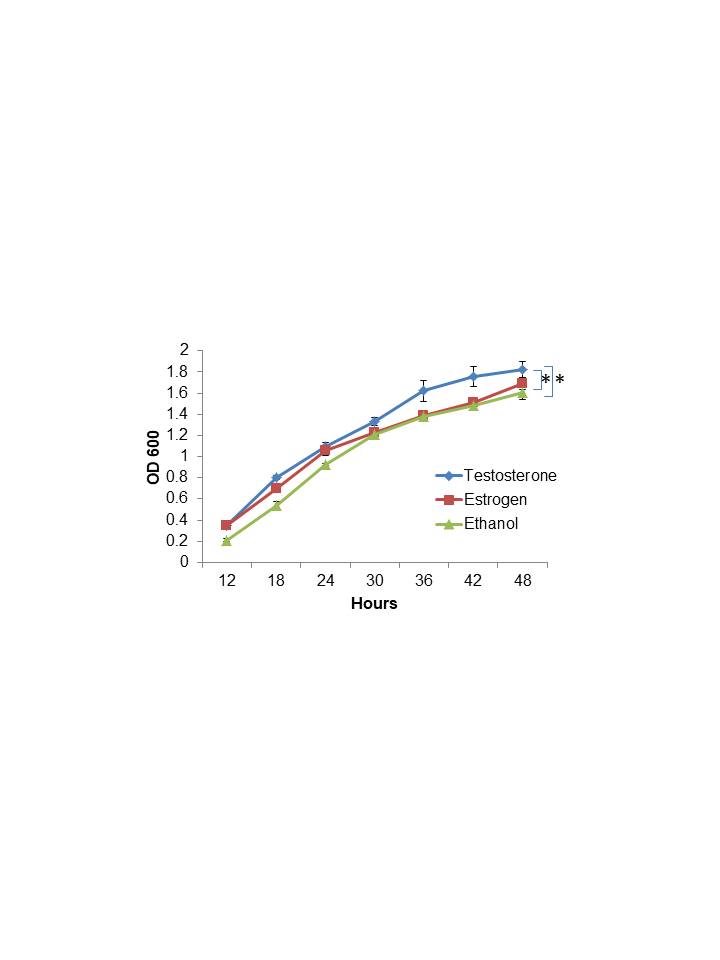


Supplementary Figure 2. Growth of H99S in L-DOPA media supplemented with steroid hormones. Absorbance was measured at an optical density of 600 nm. Error bars represent the standard deviation. Data is the average of three independent experiments. Statistical significance is represented as follows: **p* < 0.025 using linear regression analysis.


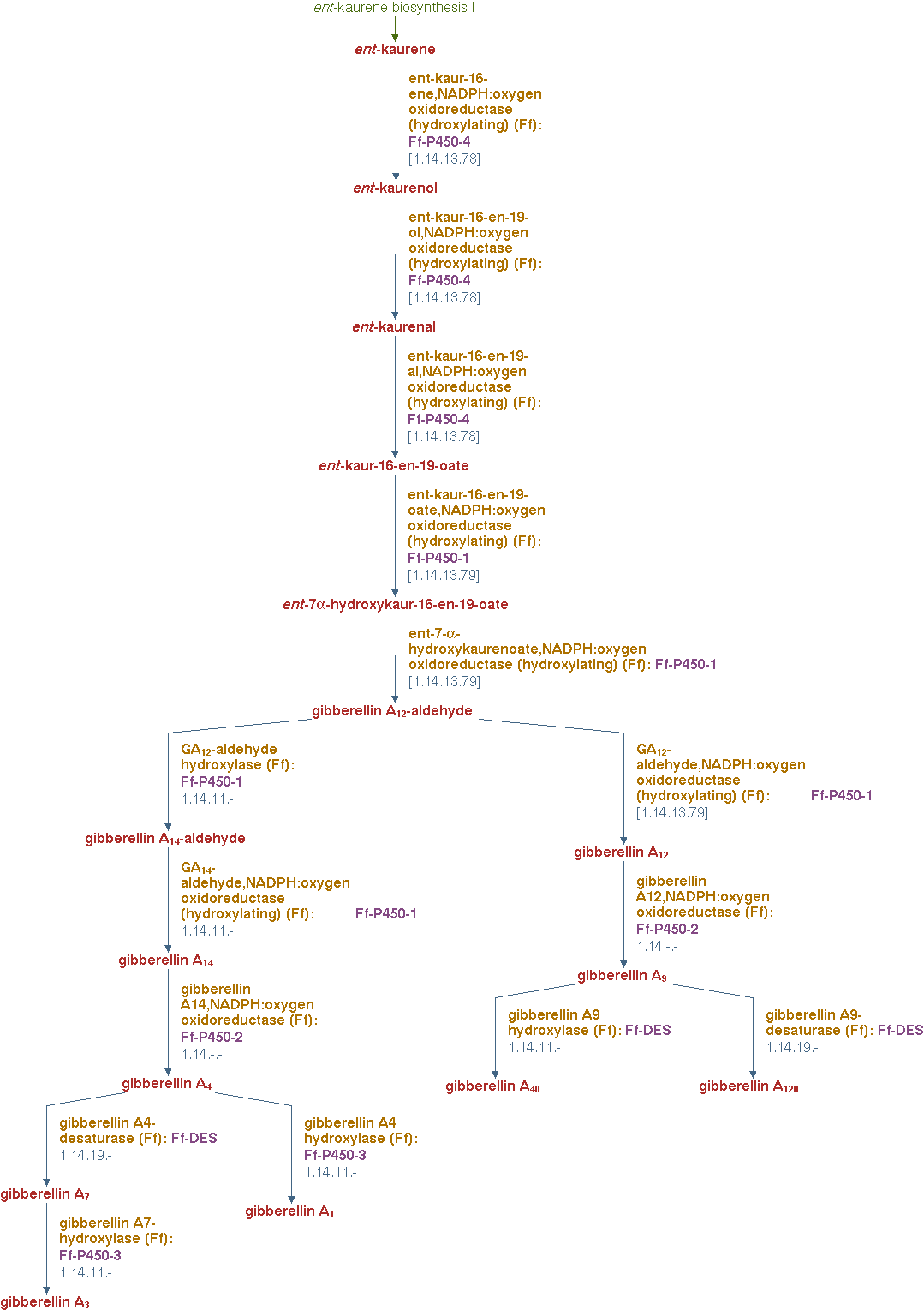


Flavonol Synthase

Supplementary Figure 3. The current GA pathway in *Gibberella fujikuroi* (adapted from the BioCyc database) (MetaCyc 2019).


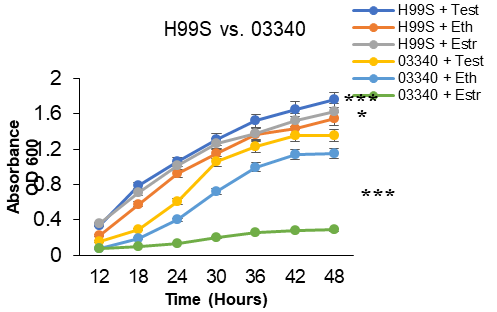

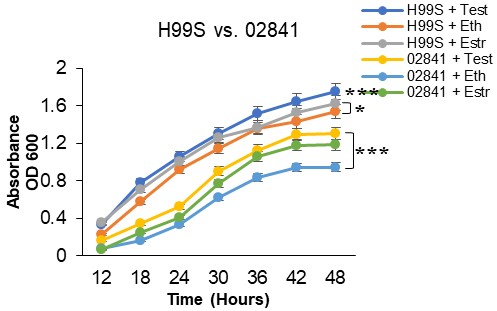

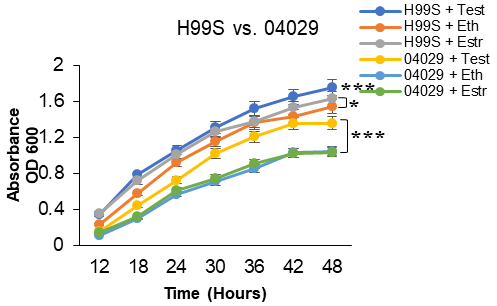

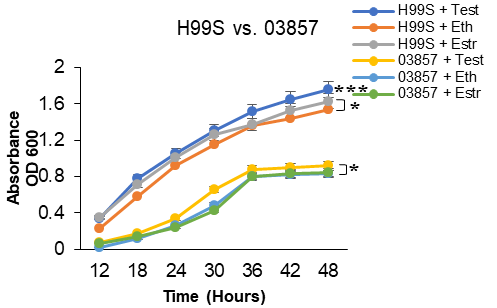

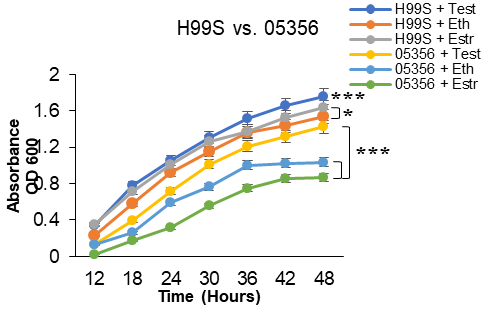


Supplementary Figure 4. Growth Curve of H99S vs. Knockout Strains (KO) in Steroid Hormone-Supplemented L-DOPA Medium. Error bars represent the standard error of the mean. Data are representative of three independent experiments. Statistical significance is represented as follows: All KO strains are significantly different from H99S+Test, ****p* < 0.0001. ****p* < 0.0001 comparing H99S+Test to H99S+Eth, **p* = 0.025 comparing H99S+Eth to H99S+Etsr. **KO Strains**: ****p* < 0.0001 comparing 03340+Test to both 03340+Estr and Eth and 03340+Estr to 03340+Eth. ****p* < 0.0001 comparing 02841+Test to 02841+Eth. ****p* < 0.00047 comparing 04029+ Test to both 04029+Estr and Eth. ****p <* 0.0001 comparing 05356+Test to 05356+Estr and 05356+Estr to 05356+Eth. **p* = 0.01 comparing 03857+Test to 03857+Estr using linear regression. Abbreviations: Test; Testosterone, Estr; Estrogen (Estradiol), Eth; Ethanol.

References

McInnes, A G, D G Smith, R C Durley, R P Pharis, G P Arsenault, J MacMillan, P Gaskin, and L C Vining. 1977. “Biosynthesis of Gibberellins in *Gibberella fujikuroi*. Gibberellin A47.” *Canadian Journal of Biochemistry* 55: 728–35. https://doi.org/10.1007/s002530100701.

MetaCyc. 2019. “Gibberellic Acid Pathway.” 2019.
